# Translation, Cross-Cultural Adaptation, and Validation of the Chronic Rhinosinusitis Patient-Reported Outcome (CRS-PRO) into Hebrew

**DOI:** 10.3390/jcm14072347

**Published:** 2025-03-29

**Authors:** Tomer Boldes, Rabie Shehadeh, Eitan Shavit, Firas Kassem, Benny Nageris, Leigh J Sowerby, Ameen Biadsee

**Affiliations:** 1 Department of Otolaryngology-Head and Neck Surgery, Meir Medical Center, Kfar Saba 4428164, Israel; rshehadeh1@gmail.com (R.S.); eitanshavit10@gmail.com (E.S.); kassemfiras@gmail.com (F.K.); bennyn@clalit.org.il (B.N.); aminbiadsee@tauex.tau.ac.il (A.B.); 2 School of Medicine, Faculty of Medical and Health Sciences, Tel Aviv University, Tel Aviv 6997801, Israel; 3 Department of Otolaryngology-Head and Neck Surgery, Western University, London, ON N6A 3K7, Canada; leigh.sowerby@sjhc.london.on.ca

**Keywords:** chronic rhinosinusitis, quality of life, patient-reported outcome measure, FESS, SNOT-22, CRS-PRO

## Abstract

**Backgrounds:** Designed to measure symptoms and quality-of-life impacts, the chronic rhinosinusitis patient-reported outcome (CRS-PRO) questionnaire is a novel instrument tailored to CRS patients. This study aimed to translate the CRS-PRO into Hebrew, adapt it cross-culturally, and assess its reliability and validity. **Methods:** A prospective study was conducted with 127 participants across three groups: CRS, functional endoscopic sinus surgery (FESS), and control groups (healthy individuals). Participants completed the Hebrew CRS-PRO at baseline and one month later. The Hebrew version was developed according to the International Society for Pharmacoeconomics and Outcomes Research guidelines for translation and cross-cultural adaptation. **Results:** Of the 127 participants (mean age 47.3 ± 17.7 years, range 18–93), 77 were males (60.6%), and 50 were females (39.4%). The Hebrew CRS-PRO demonstrated high internal consistency (Cronbach’s alpha 0.936) and strong discriminant validity among the three groups. Baseline mean scores were 7.2 for the control group, 25.2 for the FESS group, and 27.1 for the CRS group, which subsequently decreased to 6.5, 12.9, and 20.4, respectively, after one month (ANOVA, *p* < 0.001). Test–retest reliability, supported by Pearson’s correlation (*p* < 0.01) and intraclass correlation (*p* < 0.0001), demonstrated the questionnaire’s effectiveness in identifying CRS-related symptoms and monitoring improvement after FESS. **Conclusions:** The adaptation and validation of the CRS-PRO into Hebrew resulted in a reliable instrument in patients with CRS. It exhibited robust reliability, internal consistency, and strong discriminant validity, effectively differentiating between healthy individuals and CRS patients and those who are pre- and post-FESS. Additionally, the Hebrew CRS-PRO questionnaire may be effective for evaluating patients before and after FESS surgery.

## 1. Introduction

Chronic rhinosinusitis (CRS) is a persistent inflammatory condition affecting the paranasal sinuses (lasting ≥12 weeks) [1], with recent epidemiological data indicating a global prevalence of approximately 5% to 12% [2,3]. CRS is associated with multiple adverse health effects, including fatigue, depression, and a significant reduction in quality of life, with symptoms often lasting for several years. Moreover, CRS imposes a substantial societal burden, with an annual absenteeism rate of approximately 24.6 days per year and an estimated productivity loss of around USD 10,077 per patient [4]. In the United States, CRS is estimated to incur roughly USD 8.6 billion in direct healthcare costs annually, and total costs (including indirect productivity losses) may exceed USD 60 billion per year [5].

CRS significantly impacts individuals in Israel, with studies highlighting its prevalence and associated health implications. A study conducted within the Israeli Defense Forces revealed that 26.1% of patients with rhinitis and 23.3% of those with CRS developed asthma over an average follow-up of eight years, compared to only 3.7% in the control group. Additionally, these patients exhibited an increased loss of productivity and higher medical service utilization, underscoring the substantial burden of CRS on young adults in Israel [6]. Moreover, CRS patients had a 7.7-fold greater odds of developing asthma compared to healthy individuals, reinforcing the strong association between upper and lower airway diseases.

First-line treatment typically includes intranasal corticosteroid sprays and saline irrigation. However, more severe or refractory cases often require surgery [7,8,9].

In recent years, CRS management has further evolved with the introduction of biologic therapies for severe cases (targeting Type 2 inflammation in patients with nasal polyps) and the refinement of surgical techniques (e.g., balloon sinus dilation, steroid-eluting sinus stents) [10]. These advancements underscore the need for precise outcome measures, as treatment success is increasingly defined by symptom relief and quality-of-life improvement [11].

Patient-reported outcome measures (PROMs) are tested and validated tools designed to assess patients’ perspectives on disease impact and treatment effectiveness. These tools are essential components of clinical decision-making and disease management. Consequently, many international guidelines and rhinology societies support using PROMs in daily practice [8,9,12,13]. A 2015 systematic review identified 15 different PROMs validated for adult CRS [8]. Studies show that the majority of clinicians incorporate PROMs into their clinical management. Notably, a recent study reported that 61.4% of clinicians use questionnaires to evaluate CRS symptoms [14,15].

The 22-item Sino-Nasal Outcome Test (SNOT-22) is the most widely used questionnaire for assessing CRS-related symptoms and disease burden. Despite its translation into multiple languages, SNOT-22 has drawbacks, primarily because it was developed prior to the updated CRS criteria and lacks direct input from clinically confirmed CRS patients [16,17,18]. Time constraints also limit the practicality of extensive PROMs such as SNOT-22 [15,19]. The CRS patient-reported outcome (CRS-PRO), a newly developed 12-item tool, was developed following the U.S. Food and Drug Administration (FDA) PROM and offers a validated alternative for both medical and surgical CRS management [17,18]. By incorporating current CRS diagnostic criteria and patient input in its development, the CRS-PRO aims to address the limitations of earlier PROMs and provide a more streamlined, patient-centered assessment. It should be noted that SNOT-22 includes some items that may be less pertinent to today’s CRS patients or overlap in concept. For instance, it has multiple questions related to sleep (trouble falling asleep, waking up tired, etc.) and combines emotional symptoms (“frustrated/restless/irritable” are a single item). It also addresses issues such as sadness, embarrassment, or dizziness which, although possible in CRS, were not frequently reported as major concerns by patients during the CRS-PRO development. These factors lead to a disproportionate weighting of certain domains in SNOT-22 and inclusion of low-yield items (e.g., “sneezing” which most CRS patients did not rate as a top problem) [17].

Another consequence is that SNOT-22’s development process (lacking documented patient input under current standards) may not meet strict regulatory criteria for use as a primary endpoint in trials, even though it remains a valuable clinical tool.

One critical reason for adapting and validating questionnaires across different cultures and languages is to ensure that items remain conceptually equivalent and retain their original intent in varied populations. This process is especially relevant for chronic rhinosinusitis (CRS), whose symptom expression can be influenced by distinct cultural, environmental, and linguistic factors. By adhering to established cross-cultural adaptation frameworks—such as the ISPOR guidelines or the COSMIN checklist—researchers can achieve more accurate assessments of disease burden and treatment outcomes [20]. This approach prevents the potential misinterpretation of survey items and bolsters the reliability and validity of PROMs in new contexts, ultimately facilitating clearer comparisons of CRS interventions on a global scale.

CRS-PRO was created to balance these considerations and improve upon certain shortcomings of earlier PROMs. With 12 items, it is shorter than SNOT-22, aiming to cover the “sweet spot” of key CRS symptoms without irrelevant content [21]. CRS-PRO’s content was extracted from current CRS patient input, including both CRSwNP and CRSsNP populations, ensuring that it targets issues patients actually report. Because its development followed best practices (e.g., the documentation of patient-driven item generation and reduction), CRS-PRO fulfills the criteria for formal acceptance in clinical trials, which older tools like SNOT-22 have not achieved [21].

The process of translating, adapting, and validating questionnaires across different languages is essential to maintaining their accuracy and reliability in diverse populations. The primary objective of this study was to perform the translation and validation of the CRS-PRO questionnaire into Hebrew.

A secondary objective involved evaluating the tool’s validity and measurement consistency in Hebrew-speaking CRS populations receiving medical therapy or undergoing endoscopic sinus surgery (ESS).

## 2. Methods

### 2.1. Ethical Considerations

The study was approved by the Meir Medical Center Institutional Review Board (IRB# MC-23-0082; 26 March 2023), and. all participants provided written informed consent prior to enrolment.

### 2.2. Cross-Cultural Adaption

**Figure 1:**
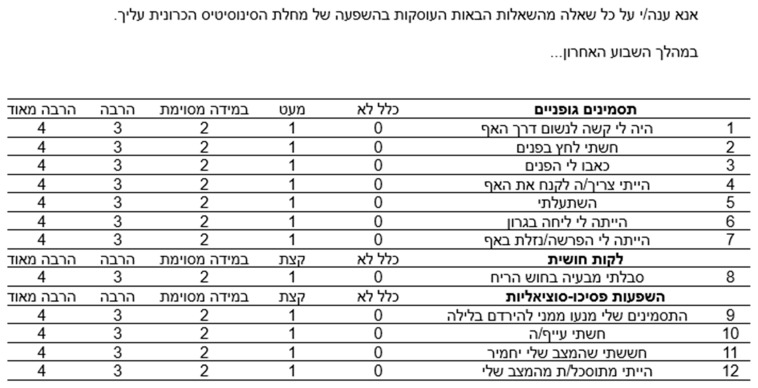
The Hebrew version of CRS-PRO.

The CRS-PRO comprises 12 questions, each scored on a five-point scale, organized into three domains: (i) physical symptoms (7 questions), (ii) sensory impairment (1 question), and, lastly, (iii) psychosocial effects (4 questions). Figure 1 shows the final Hebrew version of the CRS-PRO.

The translation and cultural adaptation of the CRS-PRO questionnaire into Hebrew was conducted in accordance to the International Society for Pharmacoeconomics and Outcome Research (ISPOR) Task Force guidelines and principles for PROMs [22]. Initially, two independent translators produced separate Hebrew versions of the original English questionnaire. These versions were then merged into a single Hebrew version, and this was translated into English by a different, independent translator. Finally, two independent bilingual reviewers evaluated the retranslated version to confirm that it accurately reflected the original content and intent of the CRS-PRO.

### 2.3. Participants

This prospective study included three groups of patients: (1) patients diagnosed with CRS (both with and without polyposis) who visited an outpatient otolaryngology clinic, (2) patients with CRS admitted for functional endoscopic sinus surgery (FESS), and (3) a control group consisted of healthy patients without a history of CRS, prior nasal surgeries, or rhinologic complaints.

Inclusion criteria required participants to be adults over the age of 18, native Hebrew speakers capable of comprehending and responding to the questionnaire, and being able to complete the questionnaire again after one month. Participants in the CRS and FESS cohorts fulfilled the diagnostic criteria for chronic rhinosinusitis outlined in the European Position Paper on Rhinosinusitis and Nasal Polyps 2020 (EPOS2020) [9]. The control group consisted of healthy individuals without a history of CRS or prior rhinologic surgery, meeting all inclusion criteria except for sinonasal pathology.

Participants were excluded if they had a prior history of nasal surgery, any sinonasal condition unrelated to CRS (with or without polyps), cognitive impairment, difficulty completing the questionnaires, or were pregnant or breastfeeding.

### 2.4. Statistical Analyses

The dataset was analyzed using SPSS (v29.0, IBM Corp., Armonk, NY, USA). Descriptive statistical methods, such as frequency distribution, percentage calculations, mean values, standard deviations, and ranges, were employed to summarize participant characteristics and symptom patterns.

Group differences in demographic characteristics and CRS-PRO scores were evaluated using Chi-square tests for categorical variables and one-way ANOVA for continuous measures.

To validate the CRS-PRO questionnaire and assess its subdomain structure, the following measures were applied:

- Internal consistency was evaluated using Cronbach’s alpha, with values above 0.70 considered indicative of an acceptable reliability;
- Test–retest reliability was assessed using the intraclass correlation coefficient (ICC) and Pearson’s correlation between initial and follow-up scores, with ICC values above 0.75 indicating good reliability, while values exceeding >0.90 were considered excellent. Pearson correlation coefficients were interpreted as follows: >0.7 (strong), 0.5–0.7 (moderate), and <0.5 (weak);
- Concurrent validity and the performance of the CRS-PRO were assessed across three subgroups: FESS, CRS, and control.

A *p*-value of less than 0.05 was considered statistically significant.

## 3. Results

### 3.1. Study Population

This prospective study was conducted between August 2023 and March 2024, enrolling 127 participants (mean age 47.3 ± 17.7 years, range 18–93). Of these, 77 (60.6%) were male. Table 1 outlines the baseline demographic and clinical characteristics. A greater proportion of males were in the FESS group (73.2%) compared to the control and CRS groups (*p* = 0.045). No statistically significant differences in age were observed between the groups (*p* = 0.119).

### 3.2. Symptom Severity Across Groups

At baseline (test), the mean total CRS-PRO scores were lowest in the control group (7.2 ± 7.6) and highest in the FESS and CRS groups (25.2 ± 12.4 and 27.1 ± 11.6, respectively, Table 2). The ANOVA results indicated a significant variation between the three cohorts (*p* < 0.001). Subsequent post hoc testing indicated that both the FESS and CRS groups had a significantly higher symptom severity than controls across all CRS-PRO domains (physical, sensing, and psychologic) as depicted in Table 2. After one month (retest), total CRS-PRO scores decreased to 6.5 ± 6.5 for controls, 12.9 ± 11.2 for FESS, and 20.4 ± 11.8 for CRS (ANOVA, *p* < 0.001). Notably, CRS-PRO test scores for the control group remained consistently low, indicating stable responses among healthy individuals.

In contrast, the FESS group demonstrated a marked reduction in scores from baseline across all domains, suggesting the questionnaire’s sensitivity to post-surgery improvements. In the CRS group, however, retest scores remained significantly higher, which reflects the persisting of symptoms in the absence of surgical intervention.

### 3.3. Internal Consistency and Reliability Analysis

An adequate internal consistency was verified using Cronbach’s coefficient alpha, which exceeded 0.70 in all groups and time points, as shown in Table 3. In particular, Cronbach’s alpha values in the CRS and FESS groups approached or surpassed 0.90, indicating excellent internal consistency.

The Cronbach’s alpha values were 0.919 for CRS, 0.924 for FESS, and 0.936 for the full study cohort.

Test–retest reliability was employed using both the intra-class correlation coefficient (ICC) and Pearson’s correlation coefficient for the total score and each domain. Pearson’s correlation coefficients between test and retest scores ranged from moderate to strong (r = 0.331–0.818, *p* < 0.01, Table 3). ICC values above 0.75 indicate good reliability, while those greater than 0.90 are deemed excellent. The ICCs further supported good to excellent reproducibility, especially in the CRS group (ICC = 0.756, *p* < 0.0001) and in the total cohort (ICC = 0.746, *p* < 0.0001).

## 4. Discussion

### 4.1. Key Findings

Our prospective study presents a successful cross-cultural adaptation and validation of the CRS-PRO questionnaire into Hebrew, following the ISPOR guidelines. Limited research has focused on translating and adapting this questionnaire from English into other languages [23,24,25].

This study yielded a Cronbach’s alpha of 0.936, indicating a strong internal consistency, slightly exceeding the values reported for the original English version (Cronbach’s alpha of 0.86) [17].

Additionally, we found a high level of ability to distinguish between groups of the CRS-PRO questionnaire (ANOVA, *p* < 0.001). Notably, the Hebrew CRS-PRO was applicable to a wide age range (18–93 years), reflecting its adaptability to adult populations., although clinicians should remain attentive to potential cognitive limitations in older adults.

### 4.2. Reliability and Validity Analyses of the Hebrew CRS-PRO

Both Cronbach’s alpha and test–retest reliability analyses confirmed that the Hebrew CRS-PRO is a stable and internally consistent tool. Pearson’s correlation coefficients (r = 0.331–0.818) and ICC values (≥0.75) indicated good to excellent reproducibility, aligning with previous Chinese and Arabic validation studies of CRS-PRO, which also reported high Cronbach’s alpha values (0.813 and 0.970, respectively) [24,25].

Our finding showed a high discriminant validity observed via ANOVA (*p* < 0.001), confirming that the Hebrew version effectively distinguishes between healthy individuals and those with CRS, as well as between pre- and post-FESS states, reflecting the questionnaire’s sensitivity to clinical change. Additionally, the control group scores remained low from test to retest (7.2 ± 7.6 to 6.5 ± 6.5), confirming good stability in healthy individuals.

The magnitude of symptom improvement captured by the CRS-PRO in post-surgery patients is consistent with that reported in other CRS outcome studies. For instance, patients in our FESS subgroup showed a substantial decrease in mean CRS-PRO scores (from 25.2 pre-operatively to 12.9 one month postoperatively), paralleling the significant improvements in quality of life seen after sinus surgery in the literature [26]. A recent meta-analysis of 40 cohorts found a mean improvement of ~24 points in SNOT-22 scores at ~10 months after endoscopic sinus surgery [26], which is comparable to the degree of postoperative change observed in our cohort. This concurrence supports the ability of the CRS-PRO to sensitively reflect clinical improvements.

As new treatment modalities gain traction [10,11], having a validated PROM like the CRS-PRO is invaluable to objectively quantify symptom changes and patient benefits that might not be fully captured by objective measures alone. For example, biologics might significantly improve a patient’s sense of smell or daily functioning, even if endoscopic appearance changes are modest—changes that a sensitive PROM would detect. Similarly, assessing outcomes of balloon dilation or drug-eluting stents should include patient-centered metrics, to ensure these techniques truly translate into improved patient well-being.

For example, we highlight Book et al. 2023 [27], conducted at a tertiary medical center in Israel, which assessed the real-world impact of biologic treatments for severe CRSwNP focused on clinical improvements using SNOT-22. Meanwhile, our study highlights the potential role of CRS-PRO as a targeted, patient-centered assessment tool for Hebrew-speaking populations. CRS-PRO incorporates direct patient input and reflects current diagnostic standards, making it a valuable addition to clinical and research settings in Israel.

It should be noted that while our study already provides robust statistical validation through Cronbach’s alpha, ICC, and ANOVA, additional assessments such as sensitivity analysis and effect size comparisons could further solidify the tool’s clinical relevance. Future studies should include effect size calculations to quantify the size of differences between CRS-PRO and SNOT-22 responses, particularly in the context of RHINO and FESS treatment outcomes.

### 4.3. Future Research Directions and Clinical Implications

Our study validates the Hebrew version of CRS-PRO, but further research is warranted to maximize its utility and to continue improving CRS management. Future studies should compare the CRS-PRO with existing PROMs (such as SNOT-22) in head-to-head assessments to determine their relative sensitivity, responsiveness to change, and patient preference. It would be valuable to investigate whether using CRS-PRO longitudinally in clinics can aid in clinical decision-making—for example, by helping to identify patients who failed medical therapy and might need further intervention. Additionally, long-term and comparative effectiveness studies are needed to inform optimal treatment strategies in CRS. Key questions include how new biologic therapies stack up against ESS over the long term, and which patients should receive which treatment first. The high cost of biologics (estimated at USD 10,000–USD 40,000 per year) versus the one-time but potentially repeatable cost of surgery (~USD 5000–USD 15,000 per procedure) highlights the need for cost-effectiveness analyses and biomarkers to guide therapy selection [28]. Future research priorities should include refining diagnostic tests to identify targetable endotypes, validating treatment protocols, and advancing therapeutic options for non-Type 2 CRS. Addressing these research areas could significantly advance personalized care—for instance, by tailoring treatment plans based on a patient’s inflammatory profile and likely response. In terms of patient-reported outcomes, future work may explore integrating electronic CRS-PRO assessments into telemedicine or patient portals, enabling real-time monitoring and early intervention for worsening symptoms.

The integration of CRS-PRO with electronic health records and telemedicine platforms could enhance its accessibility and usability in clinical practice. By incorporating CRS-PRO into digital patient management systems, clinicians could systematically track symptom progression and treatment responses in real-time.

Notably, the recent validation of the Arabic CRS-PRO by Biadsee et al. 2025 [24] further underscores the cross-cultural reliability and adaptability of the CRS-PRO instrument. Similar to our findings, the Arabic version demonstrated excellent internal consistency (Cronbach’s alpha 0.97) and robust discriminant validity in distinguishing between CRS patients, post-FESS individuals, and healthy controls (ANOVA, *p* < 0.001). Notably, both studies observed significant post-surgical score reductions in the FESS groups (Hebrew: 25.2 to 12.9; Arabic: 32.9 to 7.7), reinforcing the questionnaire’s sensitivity to clinical improvement. These parallel outcomes across linguistically and culturally distinct populations highlight the CRS-PRO’s universal applicability as a streamlined, patient-centered tool, addressing the limitations of legacy PROMs like SNOT-22 in diverse settings [24].

The consistency between the Hebrew and Arabic validations, along with prior adaptations into Chinese [23] and French [25], strengthens the global evidence base for CRS-PRO. Such cross-cultural concordance validates the instrument’s structural integrity. By integrating CRS-PRO into routine practice, clinicians can better capture patient-centric outcomes, bridging gaps between objective metrics and lived symptom experiences across cultures [24,29].

While the results underscore its utility in clinical and research settings, certain limitations should be acknowledged. To improve the external validity of CRS-PRO among Hebrew-speaking populations, future research should include participants from multiple clinical settings across Israel, incorporating regional and demographic variations. Conducting a multicenter validation will allow us to evaluate potential differences in symptom reporting based on environmental and socioeconomic factors, ensuring the broader applicability of the instrument. The study’s relatively short follow-up period (one month) may not fully capture long-term symptom trajectories or treatment outcomes. Additionally, the sample, though diverse in age, was drawn from a single center, potentially limiting the generalizability to broader populations.

Furthermore, the Hebrew CRS-PRO fills a critical gap in patient-centered outcome measurement for Hebrew-speaking populations, offering clinicians in Israel a tool to guide treatment decisions and progress. Its integration into routine practice could enhance personalized care and facilitate collaborative comparisons, while maintaining language and cultural aspects in mind, in global CRS research. Future studies should leverage these adaptations to standardize CRS outcome measurement globally, particularly as biologic therapies and minimally invasive surgical techniques redefine treatment paradigms.

## 5. Conclusions

We found that the adaption and validation of the CRS-PRO into Hebrew is a reliable instrument in a wide variety of ages. The results highlight high internal consistency, reliability, and discriminant validity in differentiating healthy individuals from CRS patients and pre- or post-ESS. Additionally, the Hebrew version of CRS-PRO can serve as an effective instrument for assessing symptom severity, treatment outcomes, and surgical interventions in CRS.

## Figures and Tables

**Table 1:** Characteristics of CRS, FESS, and control groups.

Variable	Control (*n* = 50)	CRS (*n* = 36)	FESS (*n* = 41)	Total (*n* = 127)	*p*-Value
Sex, *n* (%)					
Female	26 (52%) ^a^	13 (36.1%) ^ab^	11 (26.8%) ^b^	50 (39.4%)	0.045 *
Male	24 (48%) ^a^	23 (63.9%) ^ab^	30 (73.2%) ^b^	77 (60.6%)	
Age, years, mean (SD)	44.0 (17.7)	46.9 (17.7)	51.7 (17.3)	47.3 (17.7)	0.119
Age group, *n* (%)					
18–40	25 (50%)	14 (38.9%)	15 (36.6%)	54 (42.5%)	0.381
41–90	25 (50%)	22 (61.6%)	26 (63.4%)	73 (57.5%)	

The percentages represent the column proportions. Differences in categorical variables (sex, age group) were tested using the Chi-square test. One-way ANOVA was used to compare continuous variables (age). Superscripts (^a,b^) indicate significant differences in post hoc tests, * *p* < 0.05 (for Chi-square test, z-test with Bonferroni correction for comparisons of column proportions, and for one-way ANOVA, Tukey HSDs to examine average differences between all pairs).

**Table 2:** Test–retest symptom scores.

Variable	Control (*n* = 50)	CRS (*n* = 36)	FESS (*n* = 41)	Total (*n* = 127)	*p* Value
Test score					
Total CRS-PRO	7.2 (7.6)	27.1 (11.6)	25.2 (12.4)	18.6 (13.9)	<0.001 §
Physical symptoms	4.1 (4.4)	15.6 (6.1)	14.7 (7.8)	10.8 (8.2)	<0.001 §
Sensing	0.3 (0.7)	2.6 (1.3)	1.9 (1.8)	1.5 (1.6)	<0.001 §
Psychologic	2.8 (3.6)	9.3 (5.3)	8.7 (4.6)	6.6 (5.4)	<0.001 §
Retest score					
Total CRS-PRO	6.5 (6.5)	20.4 (11.8)	12.9 (11.2)	12.5 (11.3)	<0.001 †
Physical symptoms	3.3 (3.7)	11.8 (6.9)	6.9 (6.9)	6.9 (6.7)	<0.001 †
Sensing	0.3 (0.8)	1.9 (1.4)	1.3 (1.6)	1.1 (1.4)	<0.001 †
Psychologic	2.8 (3.6)	6.9 (5)	4.8 (4.8)	4.6 (4.7)	<0.001 †

CRS and FESS groups were *n* using ANOVA, with results presented as mean (standard deviation). § Significant post hoc values were observed for CRS vs. control and FESS vs. control, while CRS vs. FESS differences were not statistically significant. † using post hoc tests, comparisons between groups resulted in significant statistical difference.

**Table 3:** Assessment of the CRS-PRO’s reliability.

Variable	Control (*n* = 50)	CRS (*n* = 36)	FESS (*n* = 41)	Total (*n* = 127)
Cronbach’s Alpha †				
Test physical symptoms	0.76	0.795	0.886	0.902
Test psychologic	0.808	0.91	0.859	0.906
Total Test	0.857	0.911	0.909	0.942
Retest physical symptoms	0.759	0.843	0.871	0.877
Retest psychologic	0.819	0.876	0.89	0.881
Toal Retest	0.827	0.913	0.899	0.918
All physical symptoms	0.76	0.835	0.905	0.898
All psychologic	0.812	0.9	0.892	0.899
All (test–retest)	0.844	0.919	0.924	0.936
Test–Retest Correlation (Pearson’s Correlation)				
Physical symptoms	0.641 **	0.631 **	0.447 **	0.641 **
Sensing	0.673 **	0.688 **	0.818 **	0.641 **
Psychologic	0.331 *	0.736 **	0.0551 **	0.812 **
Total CRS-PRO	0.527 **	0.0527 **	0.701 **	0.518 **
ICC ***				
Physical symptoms (F)	0.770 (4.435)	0.704 (4.368)	0.444 (2.597)	0.714 (4.394)
Sensing (F)	0.797 (4.877)	0.768 (5.383)	0.872 (9.681)	0.881 (9.289)
Psychologic (F)	0.503 (1.991)	0.801 (6.546)	0.580 (3.453)	0.726 (4.153)
Total CRS-PRO (F)	0.686 (3.124)	0.756 (5.676)	0.502 (3.171)	0.746 (4.93)

* *p* < 0.05 (2-tailed); ** *p* < 0.01 (2-tailed); *** *p* < 0.0001. † Subdomain “sensing” (Q8) contains only 1 question; thus, the Cronbach’s alpha test was not applied.

## Data Availability

Data available on request from the authors. The CRS-PRO is owned and copyrighted by, and the intellectual property of, Bruce K. Tan. Reproduced with permission from Bruce Tan. Permission for use of the CRS-PRO questionnaire was obtained by contacting Tan at btan@nm.org.
